# *Cryptococcus gattii* VGIIb-like Variant in White-Tailed Deer, Nova Scotia, Canada

**DOI:** 10.3201/eid2206.160081

**Published:** 2016-06

**Authors:** David P. Overy, Scott McBurney, Anne Muckle, Lorraine Lund, P. Jeffery Lewis, Robert Strang

**Affiliations:** Atlantic Veterinary College, University of Prince Edward Island, Charlottetown, Prince Edward Island, Canada (D.P. Overy, S. McBurney, A. Muckle, L. Lund, P.J. Lewis);; Department of Health and Wellness, Halifax, Nova Scotia, Canada (R. Strang)

**Keywords:** *Cryptococcus gattii*, cryptococcosis, VGIIb-like variant, multilocus sequence typing, fungi, Nova Scotia, white-tailed deer, Canada

**To the Editor:** *Cryptococcus gattii* is a fungal pathogen that is emerging in the Pacific Northwest of North America. In Nova Scotia, Canada, previously not recognized as a *C. gattii*–endemic area*,* a variant strain similar to VGIIb caused cryptococcosis with nasopulmonary, lymph node and central nervous system involvement in a free-ranging, yearling white-tailed deer (*Odocoileus virginianus*). The deer was found in the village of Greenwood (latitude 44.9717246; longitude −64.9341295) on July 14, 2014. The deer exhibited behavioral and neurologic abnormalities, including loss of fear of humans, ataxia, circling, high-stepping gait, torticollis, and a fixed stare. Additional clinical signs were ptyalism with frothing from the mouth and dyspnea with gurgling respiration. The animal was euthanized, and the head, lungs, heart, gastrointestinal tract, liver, and kidneys were submitted for pathologic examination.

Gross examination revealed multifocal, soft, round, expansile, pale tan masses of variable sizes that had replaced or effaced the normal architecture of the tracheobronchial lymph nodes and pulmonary parenchyma. The center of the largest lymph node mass was necrotic and filled with viscous yellow material. Similar yellow gelatinous material obliterated the right ethmoturbinates rostral to the cribriform plate. In the brain, cerebellar coning was prominent. Several small, pitted lesions with dark rims were noted in the neuropil of the thalami, superior colliculi, and hippocampus. Gross lesions were absent in the liver, kidney, and gastrointestinal tract (Technical Appendix).

Microscopically, the nasal cavity, lung, tracheobronchial lymph node, and brain lesions were similar, consisting of variably sized, cystic spaces supported by various thicknesses of well-differentiated fibrovascular septa or remaining normal parenchyma. The cystic spaces, immediately adjacent tissues, meninges, and ependyma contained variable numbers of yeast associated with a granulomatous inflammatory response or a pleocellular population of lymphocytes, plasma cells, macrophages, and neutrophils (Figure). The yeast were round to oval, 15–33 μm in total diameter, with poorly staining central portions (5–12 μm in diameter) surrounded by a pale acidophilic or basophilic capsule (5–21 μm thick), which stained positively with a mucicarmine stain. Some yeast were dematiaceous, and Fontana-Masson staining was consistent with presence of melanin. Very rarely, narrow-based budding was observed in the yeast. All aforementioned morphologic characteristics, staining affinities, and lesion distributions are consistent with an infection with fungi in the genus *Cryptococcus* (*1*,*2*).

**Figure:**
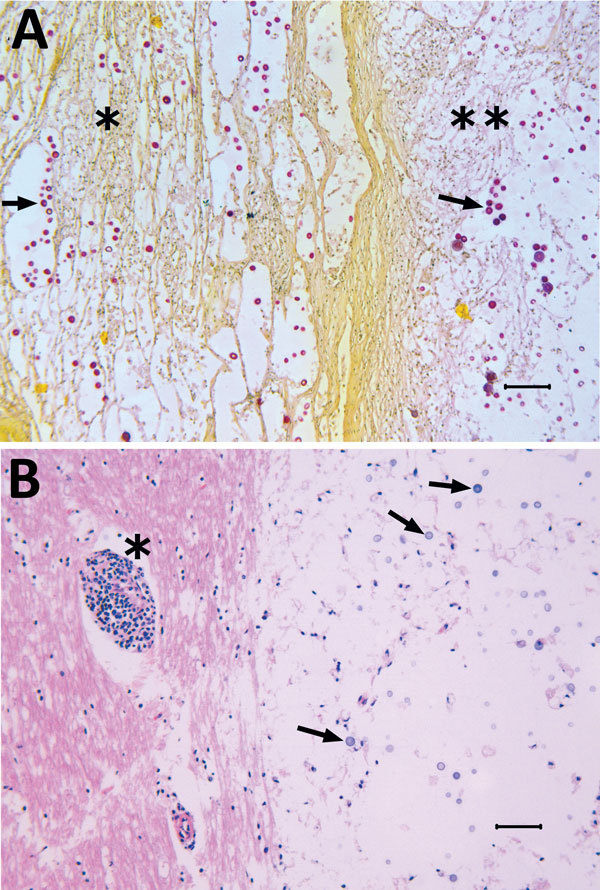
Tissue from white-tailed deer *(Odocoileus virginianus*), showing microscopic lesions caused by a unique *Cryptococcus gattii* VGIIb-like variant strain most similar to that of the VGIIb genotype; etiology was confirmed by molecular sequencing. A) Photomicrograph of lung lesions with intralesional *C. gattii* (arrows indicate examples of individual yeast) in a mass (**) and in adjacent compressed alveolar spaces (*). Mucicarmine stain. Scale bar indicates 100μm. B) Photomicrograph of a brainstem lesion with intralesional *C. gattii* (arrows indicate examples of individual yeast) and an adjacent blood vessel with a perivascular infiltrate of inflammatory cells (*). Hematoxylin and eosin stain. Scale bar indicates 50μm.

One species of *Cryptococcus* was isolated from a tracheobronchial lymph node aspirate. Matrix-assisted laser desorption/ionization time-of-flight mass spectrometry fingerprinting of the isolate (Biotyper RTC software; Bruker Daltonics Ltd, Bremen, Germany) yielded 8 diagnostic signals consistent with *C. gattii* VGIIb and VGIIc; therefore, the isolate was further classified by multilocus sequence typing based on 7 genetic loci, following the International Society for Human and Animal Mycology consensus multilocus sequence typing scheme for the *C. neoformans*/*C. gattii* species complex (*3*). Further discrimination based on allele congruence with established *C. gattii* VGII genotypes (*4*) classified the isolate as being most similar to genotype *C*. *gattii* VGIIb (CAP59 allele no. 2, GPD1 allele no. 6, LAC1 allele no. 4, PLB1 allele no. 2, URA5 allele no. 2, IGS1 allele no. 10). However, because of a slight difference in the SOD1 allele (99.5% similarity with allele no. 15), this strain is considered to be a unique variant strain, most similar to that of the VGIIb genotype. Whole-genotyping studies have provided evidence of multiple distinct introductions of the VGIIb genotype to North America (*5*). Because of the observed difference in the SOD1 allele, the VGIIb-like variant strain may represent a fourth introduction or a different VGII genotype altogether.

The white-tailed deer represents a new host species for *C. gattii* in North America. Because white-tailed deer are nonmigratory, generally exhibiting only minor seasonal movements (*6*), this infection was considered to be autochthonous, indicating endemicity of the *C. gattii* VGIIb-like variant in Nova Scotia and highlighting the value of nonmigratory animals as sentinels for emerging diseases (*7*). Incidence for this disease is highest in the Pacific Northwest, where the primary agents are *C. gattii* VGII genotypes (*2*,*4*). A pertinent literature review and consultation with regional public and veterinary health authorities determined that Québec was the most eastern province in Canada where crytococcosis associated with *C. gattii* VGII has caused clinical disease that was not potentially travel related in humans (Phillippe Dufresne, pers. comm.). In eastern North America, the *C. gattii* VGIIb genotype is reported to have caused disseminated cryptococcosis in a human in Florida, USA (*8*,*9*). Because *C. gattii* is potentially pervasive in the environment, the Nova Scotia Department of Health has alerted provincial infectious disease specialists and the provincial public health laboratory to ensure availability of the diagnostic capacity to test for the fungus. 

The *C. gattii* VGIIb genotype causes substantial, life-threatening disease in otherwise healthy hosts (*2*), and a unique VGIIb-like variant is endemic to Atlantic Canada. Therefore, continued surveillance by physicians and veterinarians in the region is warranted.

Technical Appendix Gross pathologic findings in white-tailed deer infected with *Cryptococcus gattii* VGIIb-like variant, Nova Scotia, Canada.
