# Supplementary material for: Cryptococcus gattii VGIIb-like Variant in White-Tailed Deer, Nova Scotia, Canada
Source: Emerg Infect Dis. 2016 Jun;22(6):1131–3. doi: 10.3201/eid2206.160081 (PMC4880078; doi:10.3201/eid2206.160081)
Supplement: Technical Appendix — Gross pathologic findings in white-tailed deer infected with Cryptococcus gattii VGIIb-like variant, Nova Scotia, Canada. [file 16-0081-Techapp-s1.pdf]

Article DOI: http://dx.doi.org/10.3201/eid2206.160081

Cryptococcus gattii VGIIb-like Variant in White-Tailed Deer, Nova Scotia, Canada

Technical Appendix

Gross Pathology of Infected Deer, Nova Scotia, Canada, 2014

Abundant adipose tissue in the epicardial groove, surrounding the kidneys and within the mesentery indicated the good body condition of the white-tailed deer (*Odocoileus virginianus*). Several abrasions were identified in the skin of the head which were attributed to trauma associated with the neurological signs exhibited by the deer while it was alive. Gunshot wounds were present in the head consistent with the method of euthanasia. Lesions were identified in the upper and lower respiratory tracts, tracheobronchial lymph nodes and brain. The normal architecture of the right ethmoturbinates rostral to the cribriform plate was disrupted and replaced by a yellow gelatinous material. The parenchyma of the left lateral cranioventral lung lobe was focally replaced by a soft, round, expansile, pale tan mass that measured 4.5 X 3.5 X 3.5 cm (Figure 1). On cut surface, the mass's tissue was white-tan with fine fibrous connective tissue septa dissecting through it. Similar smaller masses, ranging from 0.5 – 1.0 cm in greatest diameter, were randomly distributed throughout the remaining left pulmonary parenchyma. The right lung was enlarged, and its architecture largely effaced and distorted by multifocal to coalescing similar soft masses, the largest of which was 8.0 cm in greatest diameter (Figures 2 and 3). The tracheobronchial lymph nodes were enlarged and their normal architecture was obliterated by masses similar to those described in the lung. The largest lymph node mass was 9.0 cm in greatest diameter and when sectioned, had a necrotic center filled by viscous yellow material that was aspirated in a sterile syringe and sent for microbiological culture (Figure 4). There was prominent cerebellar coning. Several small, pitted lesions (0.5 – 2 mm in diameter) with dark rims were noted in the neuropil of the thalami, superior colliculi and hippocampus (Figures 5 and 6). Lesions were not present in the liver, kidney or gastrointestinal tract. Histology confirmed that the gross lesions described above were caused by a *Cryptococcus* sp..

Microbiological culture and molecular typing confirmed the etiology as a unique *Cryptococcus gattii* variant strain most similar to that of the VGIIb genotype.

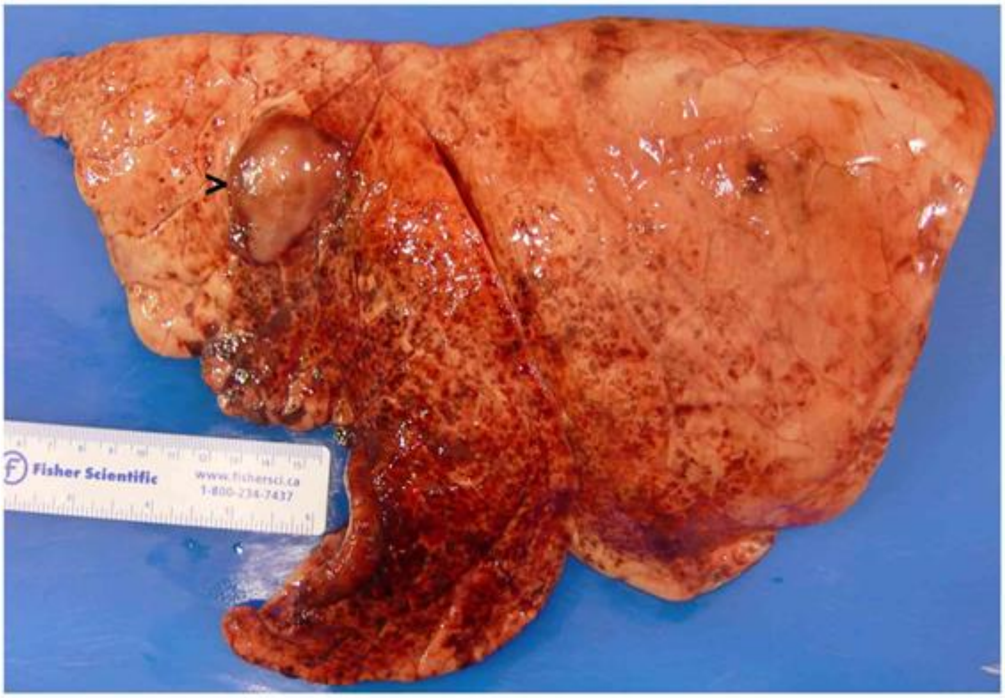

Technical Appendix Figure 1. Nodular fungal lesion in left lung of a white-tailed deer (arrow head).

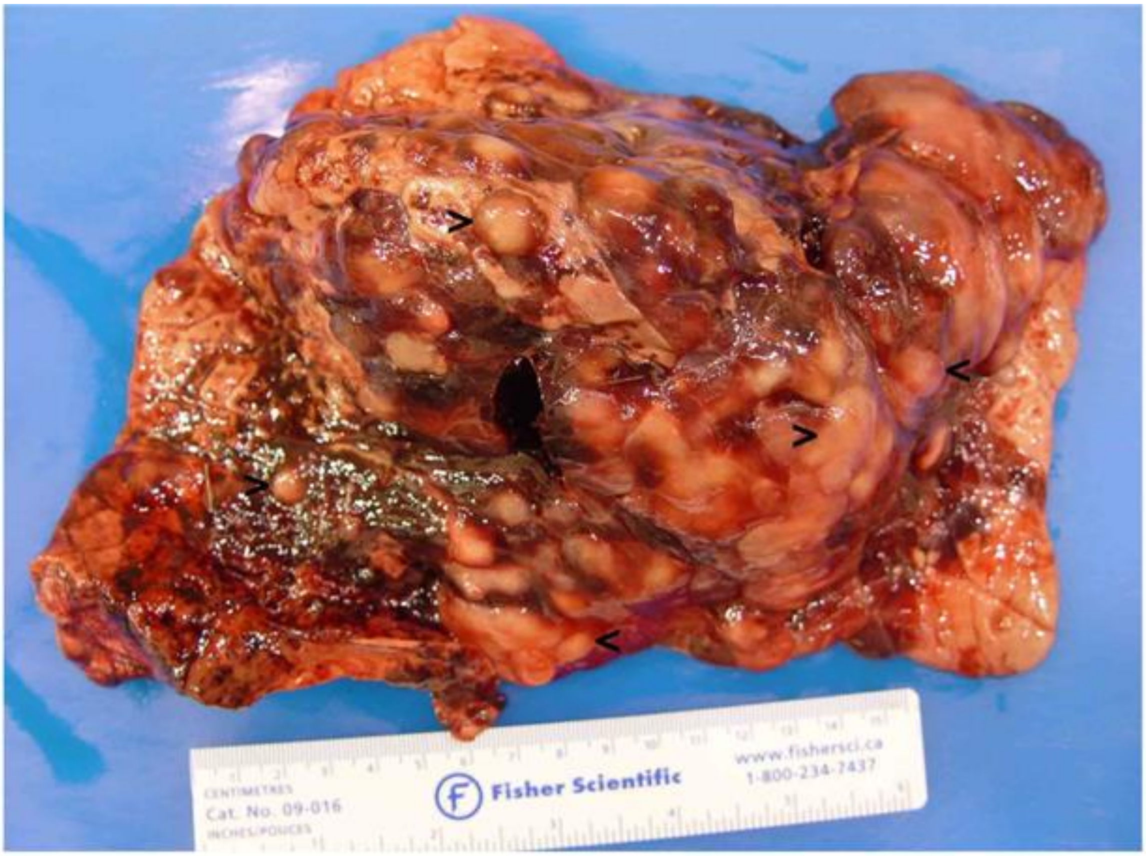

Technical Appendix Figure 2. Multifocal to coalescing nodular fungal lesions effacing and replacing the normal parenchyma of the entire right lung of the white-tailed deer (arrowheads indicate examples of individual nodules).

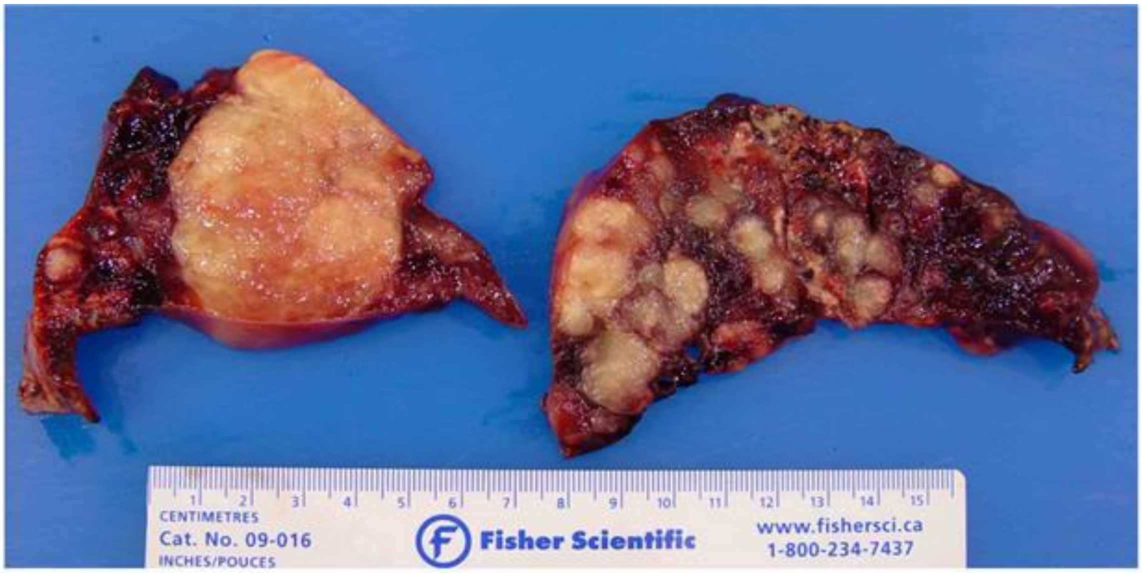

Technical Appendix Figure 3. Two cross sections of the right lung of the white-tailed deer with multifocal to coalescing, variably sized, pale tan, fungal lesions effacing and replacing the normal parenchyma.

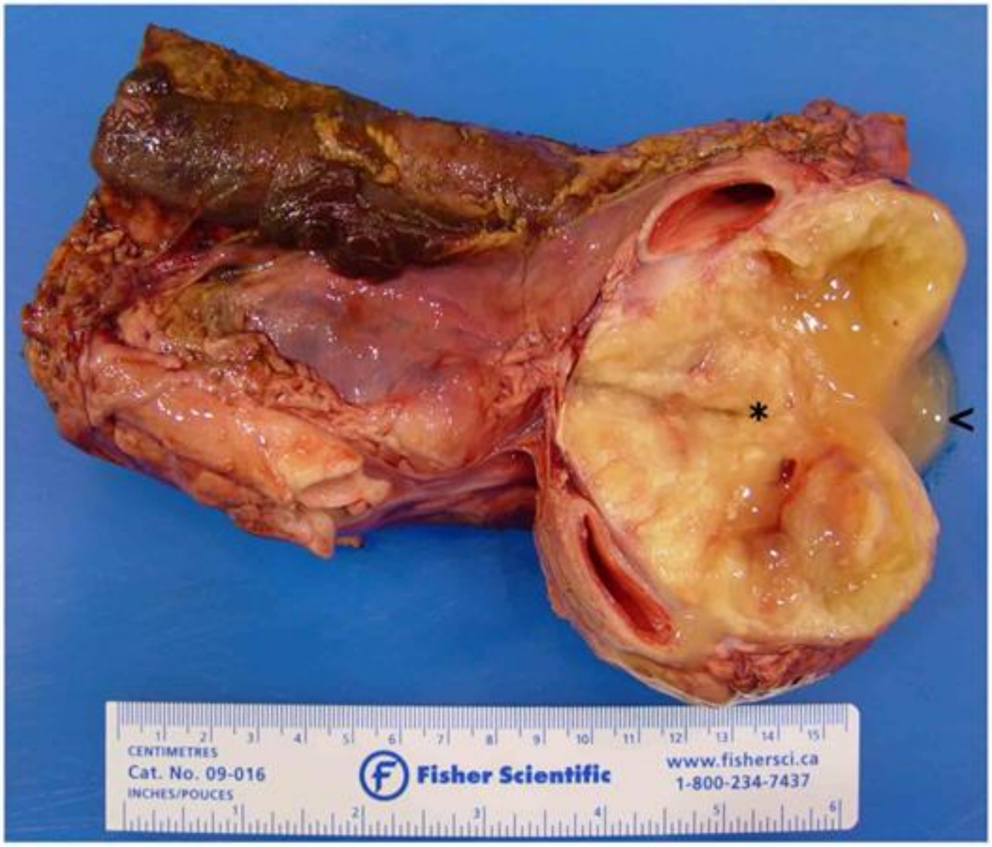

Technical Appendix Figure 4. A sectioned white-tailed deer tracheobronchial lymph node with its normal architecture replaced by a fungal lesion (asterisk). The fungal lesion has a necrotic center containing viscous yellow material (arrowhead) that was aspirated for bacteriological culture.

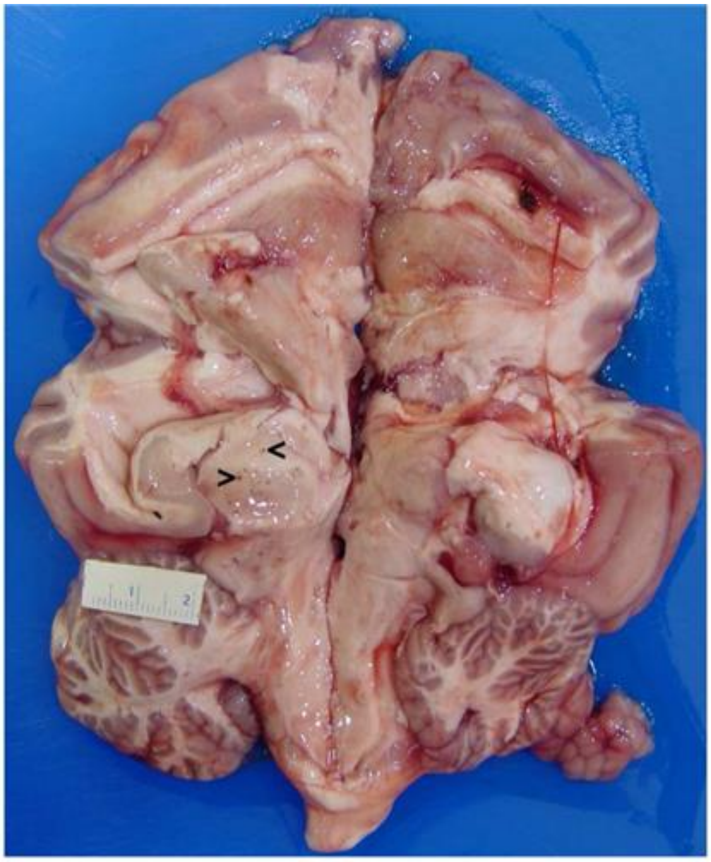

Technical Appendix Figure 5. Sagittal section of white-tailed deer brain with pitted fungal lesions in the hippocampus (arrowheads indicate examples of individual lesions) and brainstem.

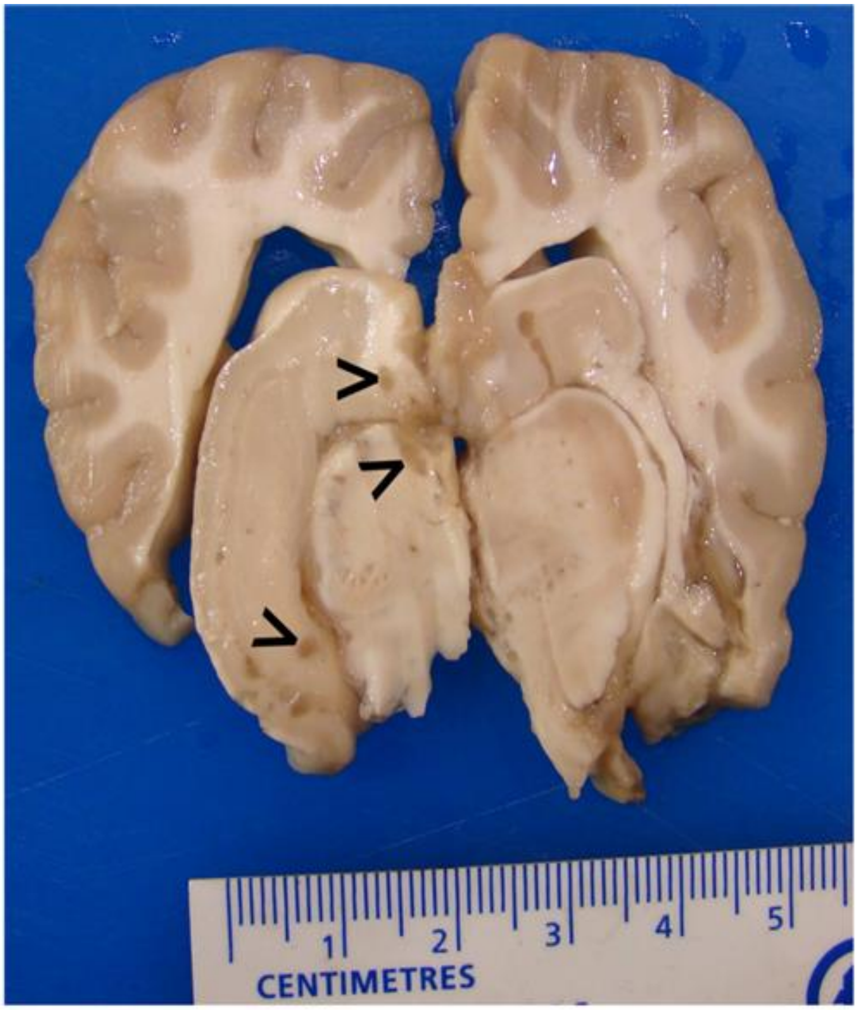

Technical Appendix Figure 6. Lesions in the hippocampus and brainstem (arrowheads indicate lesions).
